# Treadmill Training Improves Functional Recovery After Injection of Adipose‐Derived Stromal Vascular Fraction in Rats With a Spinal Cord Injury

**DOI:** 10.1002/jnr.70158

**Published:** 2026-09-20

**Authors:** Céline Ertlen, Maxime Bonnet, Mostafa Seblani, Thelma Coyle, Jean‐Michel Brezun, Nicolas Serratrice, Patrick Decherchi, Tanguy Marqueste

**Affiliations:** ^1^ Aix Marseille Univ, CNRS, ISM, UMR 7287 “Institut des Sciences du Mouvement: Etienne‐Jules MAREY,” Equipe “Plasticité des Systèmes Nerveux et Musculaire” (PSNM), Parc Scientifique et Technologique de Luminy Marseille France; ^2^ Hôpital Privé Marseille Beauregard‐Vert Coteau, Centre du Rachis Marseille France

**Keywords:** cell therapy, locomotor recovery, rehabilitation, spinal cord injury, stromal vascular fraction, treadmill training

## Abstract

Spinal cord injuries (SCI) are a major health concern, resulting in substantial functional deficits, including motor and sensory impairments around and below the affected area. Despite continuous efforts, current spinal cord repair strategies have shown limited therapeutic success. The adipose‐derived stromal vascular fraction (SVF) is a heterogeneous cell population with trophic, angiogenic, and immunomodulatory properties that has previously been shown to improve functional recovery after experimental SCI. The present study investigated whether treadmill rehabilitation could enhance the therapeutic effects of autologous SVF transplantation following severe thoracic SCI. Thirty‐six male Sprague Dawley rats were divided into four groups: NaCl (sodium chloride) (spinal cord contusion with NaCl injection), SVF (contusion with SVF autograft), NaCl‐T (contusion with NaCl injection and treadmill training), and SVF‐T (contusion with SVF autograft and treadmill training). Behavioral recovery was assessed weekly over 12 weeks using the BBB locomotor score and ladder climbing test, while gait and electrophysiological analyses were performed at the study endpoint. The combined treatment produced greater improvements in locomotor performance than either SVF transplantation or treadmill training alone, as demonstrated by higher BBB and ladder climbing scores together with improved gait parameters. Electrophysiological analyses further showed that SVF treatment restored rate‐dependent depression of the H‐reflex, reflecting improved spinal sensorimotor inhibitory function. Collectively, these findings demonstrate that treadmill rehabilitation potentiates the functional benefits of autologous SVF transplantation and supports the combination of cell therapy and rehabilitation as a promising strategy to enhance functional recovery after traumatic SCI.

## Graphical abstract

In this study, we investigated the effect of a strategy combining stromal vascular fraction (SVF) autograft with treadmill training after a spinal cord injury in rats. SVF injection was performed within the 4 h post‐injury. Analysis was performed using behavior tests (BBB and ladder climbing tests), gait analysis on a walking corridor, and electrophysiology recording (M‐ and H‐waves and physiological reflexes).

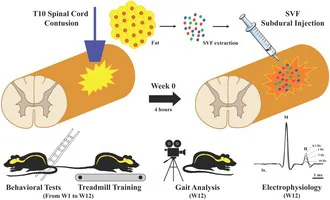

**Abbreviations**
ADSC: adipose‐derived stem cell
BDNF: brain‐derived neurotrophic factor
CPG: central pattern generator
EIF: electrically‐induced fatigue
EMG: electromyographic recording
GDNF: glial cell line‐derived neurotrophic factor
IFATS: International Federation for Adipose Therapeutics and Science
IL: interleukin
ISCT: International Society for Cellular Therapy
NaCl: sodium chloride
NSSP: normal step sequence patterns
PD: phase dispersion
PP: paw placements
RDD: rate‐dependent depression
RI: Regulatory index
SCI: spinal cord injury
SVF: stromal vascular fraction
T: training

## 1 Introduction

A spinal cord injury (SCI) can have severe consequences on bodily functions as the spinal cord is essential for the transmission of afferent and efferent neural signals between the brain and the somatic and autonomic nervous systems. SCI can be caused by various factors, including physical trauma such as road accidents, falls, sports injuries, acts of violence, or medical conditions such as tumors or infections (New and Sundararajan 2008; Algahtany et al. 2021). The functional deficits that occur following SCI depend on the severity and location of the lesion. Indeed, a SCI may induce partial or total paralysis, loss of bladder and bowel control, respiratory problems, chronic pain, or other medical complications (Sezer 2015; Hachmann et al. 2017; Sweis and Biller 2017; Biering‐Sørensen et al. 2018; Qi et al. 2018; Chamberlain et al. 2019). Furthermore SCI may reduce longevity and induce psychological disorders or the substantial financial burden of medical care (Craig et al. 2012, 2015; Middleton et al. 2012; Lo et al. 2021; Alcántar‐Garibay et al. 2022). Despite decades of experimental research aimed at reducing post‐traumatic functional deficits and repairing the spinal cord in order to preserve and restore functions, we are still unable to propose a simple and effective repair strategy that can be successfully applied to humans (Anderson et al. 2018; Flack et al. 2021). Developing an effective therapeutic strategy remains a crucial challenge in the fields of medical research and public health.

One of the main strategies that has proven effective and beneficial for patients over the past decades is activity‐based therapy (Fu et al. 2016). This strategy is built on activity‐dependent neuroplasticity (Hebb 1949; Schmidt 1985). Its primary objective is to assist patients in regaining their independence and improving their quality of life (Duan et al. 2021), particularly by restoring their hand and even walking abilities, aiming for complete autonomy. There are several training methods that are generally adapted to the specific needs of the patient, depending on the severity of the injury, the patient's physical abilities, and their recovery goals (Battistuzzo et al. 2012; Burns et al. 2017; Hicks 2021; Hillen et al. 2013). Treadmill training has already demonstrated a multitude of beneficial effects in animal models of SCI, including cats (Martinez et al. 2012, 2013; Rossignol et al. 2015) and rodents (Battistuzzo et al. 2012; De Leon and Dy 2017; Leblond et al. 2003; Martinez et al. 2013; Sandrow‐Feinberg et al. 2009). This training method, and more specifically body weight supported treadmill training (BWSTT), is the most commonly used method in clinical practice (Wernig et al. 2000). Research has demonstrated that BWSTT has the capacity to restore bladder and bowel control, significantly improve muscle activation patterns, and facilitate reorganization of the corticospinal tract in individuals who have sustained injuries. This, in turn, results in improved locomotor activity (Anwer et al. 2014; Harkema et al. 2012; Knikou 2012; Morrison et al. 2018; Protas et al. 2001; Wernig et al. 1998, 2000). Furthermore, greater functional recovery has been observed in individuals with SCI less than 12 months old, compared to patients with SCI more than 12 months old, although benefits have been observed in both groups (Zieriacks et al. 2021). In both animals and humans, the effects of treadmill training were enhanced when coupled with spinal cord repair strategies, such as the use of umbilical cord mesenchymal stem cells (Martinez et al. 2013), bone marrow stem cells (Bansal et al. 2016; Kim et al. 2018; Massoto et al. 2020; Sun et al. 2023), Schwann cells (Anderson et al. 2017; Gant et al. 2022), neural stem cells (Tashiro et al. 2016) or adipose tissue‐derived stem cells (ADSC) (Keikhaei et al. 2023; Takahashi et al. 2023).

The adipose‐derived stromal vascular fraction (SVF) is a specific component of adipose tissue that contains, after the exclusion of fat cells, various cell types. Among these cells are pre‐adipocytes, fibroblasts, adipose‐derived mesenchymal stem cells (ADSCs), endothelial progenitor cells (EPCs), pericytes, macrophages, and lymphocytes (Alexander 2012). *The International Federation for Adipose Therapeutics and Science* (IFATS) and *the International Society for Cellular Therapy* (ISCT) have quantified the cellular subpopulations within the human SVF which include 15% to 30% ADSCs, 10% to 20% EPCs and endothelial cells, 5% to 15% macrophages, 10% to 15% lymphocytes, 10% to 15% neutrophils, and 3% to 5% pericytes (Bourin et al. 2013). Furthermore, recent research indicates that this cellular composition is consistent across different sexes and anatomical sites (Tauer et al. 2022). The use of SVF presents itself as an alternative to ADSCs in clinical research, particularly because it can be isolated in a matter of minutes for immediate use, without the need for a culture step, thus offering a more practical solution for rapid action (Solodeev et al. 2023). Thanks to its regenerative, immunomodulatory, and angiogenic properties, SVF has been widely investigated in regenerative medicine and has demonstrated a favorable safety profile in several preclinical and clinical applications, including musculoskeletal (Kim et al. 2024; Onoi et al. 2023; Usuelli et al. 2018), cardiovascular (Carstens et al. 2017, 2020; Comella et al. 2016) and neurological disorders (Carstens et al. 2020; Riordan et al. 2009, Ferreira et al. 2023). More recently, our group demonstrated that immediate autologous SVF transplantation improves functional recovery and electrophysiological outcomes after thoracic spinal cord contusion in rats (Ertlen et al. 2024). Whether these therapeutic effects can be further enhanced by rehabilitation remains unknown.

In the present study, we investigated whether combining immediate autologous SVF transplantation with treadmill rehabilitation enhances functional recovery after severe thoracic SCI. To address this question, behavioral, gait, and electrophysiological assessments were performed over a 12‐week follow‐up period.

## 2 Materials and Methods

### 2.1 Animals

Experiments were conducted in 49 adult male Sprague Dawley rats, 250 to 300 g (Élevage JANVIER, Le Genest Saint Isle, France). Rats were paired and housed in plastic cages with smooth bottoms, with a 12:12‐h light/dark photoperiod and a temperature of 22°C. They had access to drinking water and rat chow (Safe, Augy, France) ad libitum. Animals were acclimated to the animal facility and behavioral devices for a period of 2 weeks prior to the start of the experiment, to reduce inter‐individual differences and optimize their performance. In our study, only male rats were used in order to avoid the influence of the estrous cycle and thereby limit the potential neuroprotective effects of female sex hormones, such as estrogen (Datto et al. 2015). This approach helps to reduce hormonal variability and increase the consistency of the results. As required by the animal‐experiment ethics committee, group sizes were determined from preliminary tests using a small number of animals, which allowed us to calculate the sample size needed to detect significant differences. A multi‐scale, multi‐modal analysis confirmed the feasibility of the study based on pre‐experiments involving three rats per group. These tests ensured a statistically adequate sample size while adhering to the 3R principle (here, Reduction).

Out of the 49 animals subjected to spinal cord contusion: 4 were excluded due to a BBB score ≥ 5 on day 1, 3 were excluded because of functional asymmetry (BBB score difference ≥ 3 between hindlimbs), 4 were euthanized due to surgical complications (≥ 20% weight loss or signs of suffering), and 2 were euthanized due to severe self‐mutilation of the hindlimbs.

These inclusion and exclusion criteria allowed us to ensure the homogeneity and reproducibility of the lesions within our experimental cohort. Thus, 36 rats were selected and continued the study until its completion.

### 2.2 Ethical Considerations

The experiments were conducted in compliance with French legislation (Decrees and orders No. 2013‐118 of February 1st, 2013, JORF No. 0032) pertaining to animal care guidelines for animal experimentation, and they were conducted after receiving approval from the Animal Care Committees of *Aix‐Marseille Université* (AMU) and *the Centre National de la Recherche Scientifique* (CNRS). The authorization number granted by the French Ministry of Higher Education, Research, and Innovation (MESRI) is APAFIS#30957. The experiments were carried out in accordance with the recommendations outlined in the Guide for Care and Use of Laboratory Animals (U.S. Department of Health and Human Services, National Institutes of Health), as well as in compliance with directives 86/609/EEC and 2010/63/EU of the European Parliament and the Council, dated 24 November 1986 and 22 September 2010, respectively. Additionally, the experiments adhered to the ARRIVE (Animal Research: Reporting of In Vivo Experiments) guidelines. All individuals involved in the experiments held the necessary licenses to conduct live animal experiments, and all experimental facilities held national authorization for animal housing (License No. B13.013.06).

Buprenorphine (0.03 mg/kg, Bruprécare Multi‐dose, Axience Santé Animale SAS, Pantin, France) was subcutaneously administered during anesthesia and after surgery daily for 3 days, and a broad‐spectrum antibiotic (Oxytetracycline, 400 mg/L, Sigma Aldrich, Saint‐Quentin Fallavier, France) was dissolved in their drinking water for 1 week as a preventive measure against infections. If necessary, manual bladder expression was performed twice daily. Then, animals were replaced in collective cages. The health condition of the animals was daily monitored with the rat grimace scale (Sotocina et al. 2011), and any animals exhibiting signs of suffering were sacrificed and excluded from the study.

### 2.3 Protocol Design and Experimental Groups

After a 2‐week familiarization program (1 h per day, 3 days per week) involving various behavioral tests, as well as treadmill running, reference values (PRE‐) for each test were measured. Then, the animals were randomly divided into four experimental groups: (1) NaCl, considered as a control group (*n* = 9): Thoracic T10 contusion induced (W0) followed by immediate NaCl injection at injury site; (2) NaCl‐T (*n* = 9): Thoracic T10 contusion induced (W0) followed by immediate NaCl injection at injury site and daily treadmill training (T) for 10 weeks (W2‐W12); (3) SVF (*n* = 9): Epididymal fat harvested, processed to extract SVF, and autologously injected at T10 contusion injury site (W0); (4) SVF‐T (*n* = 9): Epididymal fat harvested, processed to extract SVF, and autologously injected at T10 contusion injury site (W0) followed by daily treadmill training (T) for 10 weeks (W2–W12).

**FIGURE 1:**
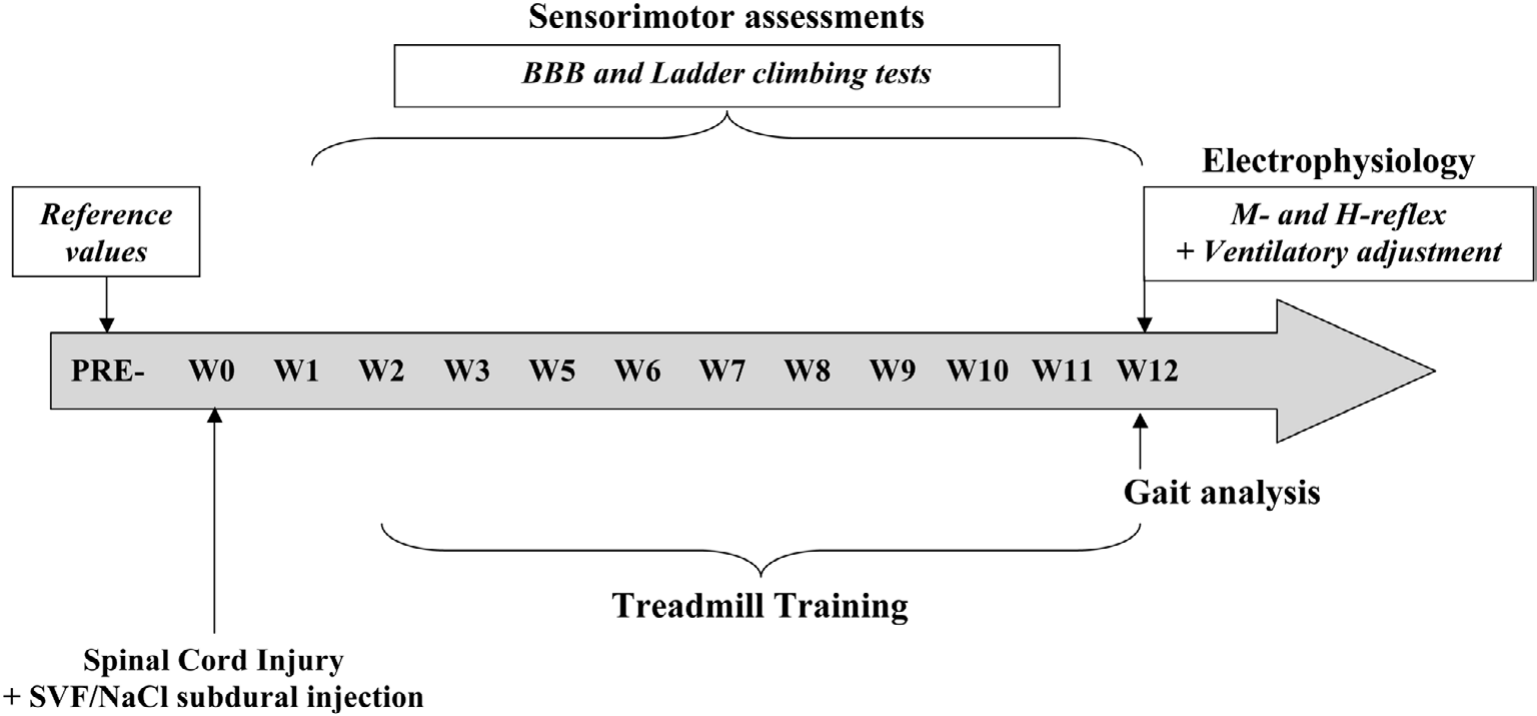
Experimental protocol. One week before surgery, baseline values (PRE‐) for each functional test were recorded. Contusions and NaCl or SVF injections were performed at W0. Thereafter, functional recoveries were assessed weekly for 12 weeks (W1 to W12) using locomotor tests (BBB and ladder‐climbing tests). Some animals have been enrolled in a training program from W2 to W12 (one 15‐min session per day, 5 days per week, at a speed varying from 15 to 30 m per minute). At the end of W12, gait analysis tests were performed, and electrophysiological recordings were performed.

Functional recovery of the hindlimbs was assessed for each animal weekly, starting from 1 week after the injury (W1), throughout the following 12 weeks using behavioral tests (BBB and ladder climbing tests). At the end of the twelfth week (W12), a gait analysis test was performed, and the functionality of the sensorimotor circuits was assessed by electrophysiological recordings of the Hoffmann reflex (H‐reflex) below the injury site and of the ventilatory frequency adjustments after inducing muscle fatigue to activate the pontobulbar ventilatory center through the metabosensitive pathway. The chronological order of our protocol is schematically shown in Figure 1.

### 2.4 Fat Removal and SVF Purification

The fat removal and SVF purification techniques were previously described (Ertlen et al. 2024). Briefly, all surgeries were performed on the animals in the SVF groups during the light cycle. Animals were initially anesthetized with isoflurane (3%–5% in oxygen) in an anesthetic chamber (Isoflo 100%, Zoetis SAS, Malakoff, France), followed by deep anesthesia through an intraperitoneal injection of Ketamine (75 mg/kg, 100 mg/mL, i.p.; Ketamidor, Axience S.A.S., Pantin, France) and Medetomidine (0.5 mg/kg, 0.85 mg/mL, i.p.; Domitor, Orion Pharma, Issy‐les‐Moulineaux, France) and buprenorphine (0.03 mg/kg). The body temperature was maintained at 37°C by a heating pad. A midline ventral incision was made to access the abdominal cavity and harvest around 1.3 ± 0.3 cm^3^ of epididymal fat, which was subsequently manually purified (François et al. 2020) to obtain the SVF. As previously observed, this fat harvesting procedure does not induce a significant inflammatory response at the spinal lesion level (Ertlen et al. 2024). The viability and the concentration of viable nucleated cells were measured in duplicate with the LUNA‐FX7 (Aligned Genetics, Logos Biosystem, France) immediately after SVF purification. Then, the purified SVF was kept at room temperature for injection within 4 h following the purification.

### 2.5 SCI and SVF Subdural Injection

The surgery procedure leading to the exposure and lesion of the spinal cord, and the technics of SVF subdural injection were detailed previously (Bonnet et al. 2020; Ertlen et al. 2024; Seblani et al. 2024). Briefly, the animals were deeply anesthetized with an intraperitoneal injection of Ketamine and Medetomidine and received an injection of buprenorphine at same concentrations as above. A midline dorsal incision was made over T8–T12 spinous processes, and the superficial muscles were retracted to expose the thoracic vertebrae. A dorsal laminectomy was performed at T10 to expose the spinal cord without damaging the dura mater. The T10 contusion was made at the midline of the spinal cord, using an IH‐0400 impactor with a 10‐g rod with a force of 200 kdyn corresponding to a severe (Scheff et al. 2003) and symmetric contusion, as shown in by Seblani et al. (Seblani et al. 2024). The presence of a visible subdural hematoma combined with the immediate onset of reflexive hindlimb spasms following the impact—reliable indicators of a massive spinal reflex discharge triggered by the contusion—confirmed that the contusion procedure should be executed correctly. Animals exhibiting these two signs must generally obtain a BBB score ≤ 5 on day 1, thereby confirming successful lesion induction. Thus, to ensure the effectiveness of the lesion, a BBB test was performed the day after the lesion was created and animals with a BBB ≥ 5 were excluded from the experiment. As previously described, the BBB score robustly reflects the anatomical extent of the lesion and tissue preservation (Basso et al. 1995; Song et al. 2018; Yan et al. 2022).

After SCI, the NaCl and NaCl‐T groups received a subdural injection of 50 μL of NaCl, and the SVF and SVF‐T groups received a subdural injection of 50 μL of autologous SVF at the lesion site. For each injected animal, a single horizontal subdural injection was performed manually at a rate of 25 μL per minute using a 50‐μL microliter syringe (Hamilton, #80500). The muscles and skin were sutured after the procedure.

After SCI, all animals exhibited severe bilateral hindlimb paralysis with limited or no joint mobility. The animals maintained a consistent body weight throughout the experimental period, indicating good overall health and nutritional status.

### 2.6 Behavioral Tests

Before surgery, baseline reference values (PRE‐) were recorded for each test. Then, over a 12‐week period, starting 1 week after the injury (W1), the BBB and ladder climbing tests were conducted once a week. At week 12 (W12), gait analysis data were collected. These assessments were conducted by two experimenters in a blinded manner, ensuring unbiased evaluations.

#### 2.6.1 BBB‐Test

The locomotor functions were assessed using the Basso–Beattie–Bresnahan (BBB) test (Basso et al. 1995). The rats were allowed to freely walk in a circular Plexiglas enclosure arena with an anti‐slip floor, measuring 95 cm in diameter and 40 cm in height. The locomotor activity was recorded for 4 min using a camcorder (GoPro Hero11) and was scored later using the BBB scale. The locomotor scores of the two hindlimbs were averaged for each rat and for each test session.

#### 2.6.2 Ladder Climbing Test

Fine motor skills and coordination were tested during climbing a 45° inclined ladder (100 × 1500 mm), with metal rungs (4 mm in diameter) spaced at equal intervals of 20 mm, with 150‐mm‐high side walls (Bonnet et al. 2020; Li et al. 2003; Semler et al. 2011). The rats were placed at the bottom of the ladder, and their climbing actions were captured using a camcorder (GoPro Hero11) positioned under the ladder to provide a ventral view of their hindlimbs. The position of the hind limbs on the rungs was scored frame by frame: 0—hind limb completely misses the rung, 1—hind limb poorly positioned but contributes to upward movement, 2—hind limb correctly positioned and contributes to upward movement. The scores from both sides were averaged, resulting in a climbing score ranging from 0 to 20. The calculated score was expressed as a percentage of the baseline score at week 0 (PRE‐).

#### 2.6.3 Gait Analysis

As previously described, motor function and coordination were quantitatively assessed using a customized gait analysis system (Ertlen et al. 2024). This system involved a glass walking plate placed in a corridor (110‐cm long, 8‐cm wide, with opaque walls 15‐cm tall) illuminated by fluorescent light, with a GoPro Hero11 camera positioned in front of the setup to record the animal's reflected footprints on the glass surface. Data collection occurred once the animals had regained a sufficient level of weight‐supported stepping (BBB score of 11 or higher) (Hamers et al. 2001). The gait analysis parameters were derived from the animals' five consecutive full steps and were analyzed using a customized MATLAB R2022b function based on the MouseWalker system (Mendes et al. 2015).

As previously outlined (Khalki et al. 2018), the following parameters were computed for each run by averaging the values:

- *Average speed*: the rate of forward movement along the runway (cm/s).
- *Stride length*: the distance between two consecutive placements of the same paw (cm).
- *Stance phase*: the duration of paw contact with the glass plate (s).
- *Swing phase*: the duration during which the paw is in the air (s).
- *Step cycle* = Stance phase + Swing phase (s).
- *Phase dispersion (PD)*: the timing between the first contacts of pairs of paws, expressed as a percentage of the step cycle of the reference paw. A PD of 0% indicates a perfect alternance between the pair of limbs. However, a PD of 50% reflects an asynchrony between the pair of limbs.
- *Step sequence patterns*: considered as normal when the animal sequentially placed its four paws in an alternate, cruciate (diagonal sequences), or rotate (lateral sequences) pattern based on the order of limb recruitment (Cheng et al. 1997) (Table 1).
- *Regulatory index (RI)*: a measure of limb coordination, expressed as a percentage of the number of normal step sequence patterns (NSSP) relative to the total number of paw placements (PP): RI = NSSP × 4/PP × 100 (%).

**TABLE 1:** Regular step patterns.

Category	Abbreviation	Sequence
Cruciate	Ca	RF‐LF‐RH‐LH
	Cb	LF‐RF‐LH‐RH
Alternate	Aa	RF‐RH‐LF‐LH
	Ab	LF‐RH‐RF‐LH
Rotate	Ra	RF‐LF‐LH‐RH
	Rb	LF‐RF‐RH‐LH

*Note:* (a) Departure of a right limb. (b) Departure of a left limb (From Cheng et al. 1997).

Abbreviations: LF, left forelimb; LH, left hindlimb; RF, right forelimb; RH, right hindlimb.

### 2.7 Treadmill Training

Two weeks after contusion and injection of either NaCl or SVF, animals in NaCl‐T and SVF‐T groups followed a quadrupedal treadmill training program based on a protocol previously described (Rossignol et al. 2015; Bonnet et al. 2020). In accordance with the 3Rs rule (especially refinement), training only began after the wounds had healed and pain relief and antibiotic treatments had been completed. Briefly, animals were trained 15 min a day, 5 days/week for 10 weeks (W2–W12) at variable speeds depending on their locomotor recovery (15–30 m/min) (Médical Développement, Saint‐Etienne, France). Initially, rats were unable to support their body weight or generate autonomous rhythmic locomotion in hind limbs. Therefore, the animal's trunk was manually supported to limit lateral imbalances, and perineal stimulation was performed to activate the sublesional central pattern generator (CPG) and induce reflex rhythmic locomotion. Stimulation consisted of tonic pinching of the perineal skin combined with gentle rubbing at 1–2 Hz, individually adjusted to trigger locomotion. Force was progressively increased at the start of each session until the rat responded, then held constant for the duration of the session, as long as it remained unable to support its weight, as has been done in previous studies (Alluin et al. 2011; Bonnet et al. 2020). As animals regained the ability to maintain body weight and generate locomotion, treadmill speed was increased in 2 m/min increments every 2 min.

### 2.8 Electrophysiological Recordings

Twelve weeks after the SCI (W12), animals were prepared for electrophysiological recordings. This involved initial sedation (isoflurane 3%–5% in oxygen), followed by deep and terminal anesthesia through intraperitoneal injection of Ketamine, Medetomidine, and buprenorphine, at the same concentrations as above. The *peroneal* nerves of the hindlimbs were isolated for stimulation, and the *Tibialis anterior* muscles were exposed for electromyographic (EMG) recording.

#### 2.8.1 M‐ and H‐Waves

The M‐ and H‐waves were recorded by stimulating the *peroneal* nerve and the decrease in reflex magnitude relative to repetition rate, also called rate‐dependent depression (RDD) of the H‐reflex, was analyzed by expressing the H_max_/M_max_ ratio obtained at stimulation frequencies of 1, 5, and 10 Hz relative to the H_max_/M_max_ ratio obtained at the baseline frequency of 0.3 Hz (Bonnet et al. 2020; Caron et al. 2016; Crone and Nielsen 1989; Ertlen et al. 2024; Gueye et al. 2015; Hultborn et al. 1996; Ishikawa et al. 1966; Lee et al. 2005; Lloyd and Wilson 1957; Nawrotek et al. 2017; Schindler‐Ivens and Shields 2000).

#### 2.8.2 Ventilatory Responses to Muscle Activation

Repetitive stimulation of *Tibialis anterior* muscles allowed measurement of the ventilatory adjustments via the activation of muscle metabosensitive afferent fibers and under regional circulatory occlusion which isolated the neural drive and abolished the hormonal communication (Decherchi et al. 2007). Thus, changes in ventilatory frequency after muscle electrically‐induced fatigue (EIF) were expressed in percent (Δcycle/min [%]) of the mean cycles recorded 2 min before muscle stimulation (Bianco et al. 2011; Bonnet et al. 2020; Caron et al. 2016; Decherchi et al. 2007; Ertlen et al. 2024; Gueye et al. 2015; Nawrotek et al. 2017; Seblani et al. 2024). Ventilatory frequency was measured by averaging over fixed intervals, and muscle fatigue was confirmed by the decay of force as previously described (Bianco et al. 2011; Decherchi et al. 2007; Gueye et al. 2015; Pertici et al. 2013).

### 2.9 Euthanasia

Following the electrophysiological recordings, according to ethical recommendations, animals were sacrificed with an overdose of pentobarbital sodium (390 mg kg^−1^, i.p., Euthasol Vet.) and the spinal cords were removed for further analysis.

### 2.10 Statistical Analysis

Statistical analyses were performed using GraphPad Prism software environment for statistical computing and graphics (GraphPad Prism Software Inc., version 8.0.2, San Diego, CA, USA).

For the analysis of BBB test, ladder test scores and H_max_/M_max_ ratios, a two‐way ANOVA (groups factor × time or stimulation frequency factor) for repeated measures was done, allowing comparison of data between groups and over time or stimulation frequency. For analysis of Catwalk test and ventilation response analysis after EIF at 12 weeks post‐injury, a one‐way ANOVA was performed (groups factor). Subsequently, a Tukey's multiple comparisons post hoc test was conducted with Bonferroni correction. The results were expressed as mean ± SEM (standard error of the mean). Statistical significance was considered when *p* < 0.05.

## 3 Results

### 3.1 SVF Characteristics

The mean volume harvested from epididymal fat for 250–300 g animals was around 1.7 ± 0.4 cm^3^. Analysis of the SVF indicated that cell viability was 93.6% ± 2.94% and that recovery yield was 1.77 ± 0.86 × 10^6^ cells per mL of fat. Furthermore, in groups treated with SVF, 10^6^ cells in the 50 μL were injected under dura matter, at the lesion site.

### 3.2 Behavioral Tests

#### 3.2.1 BBB‐Test

**FIGURE 2:**
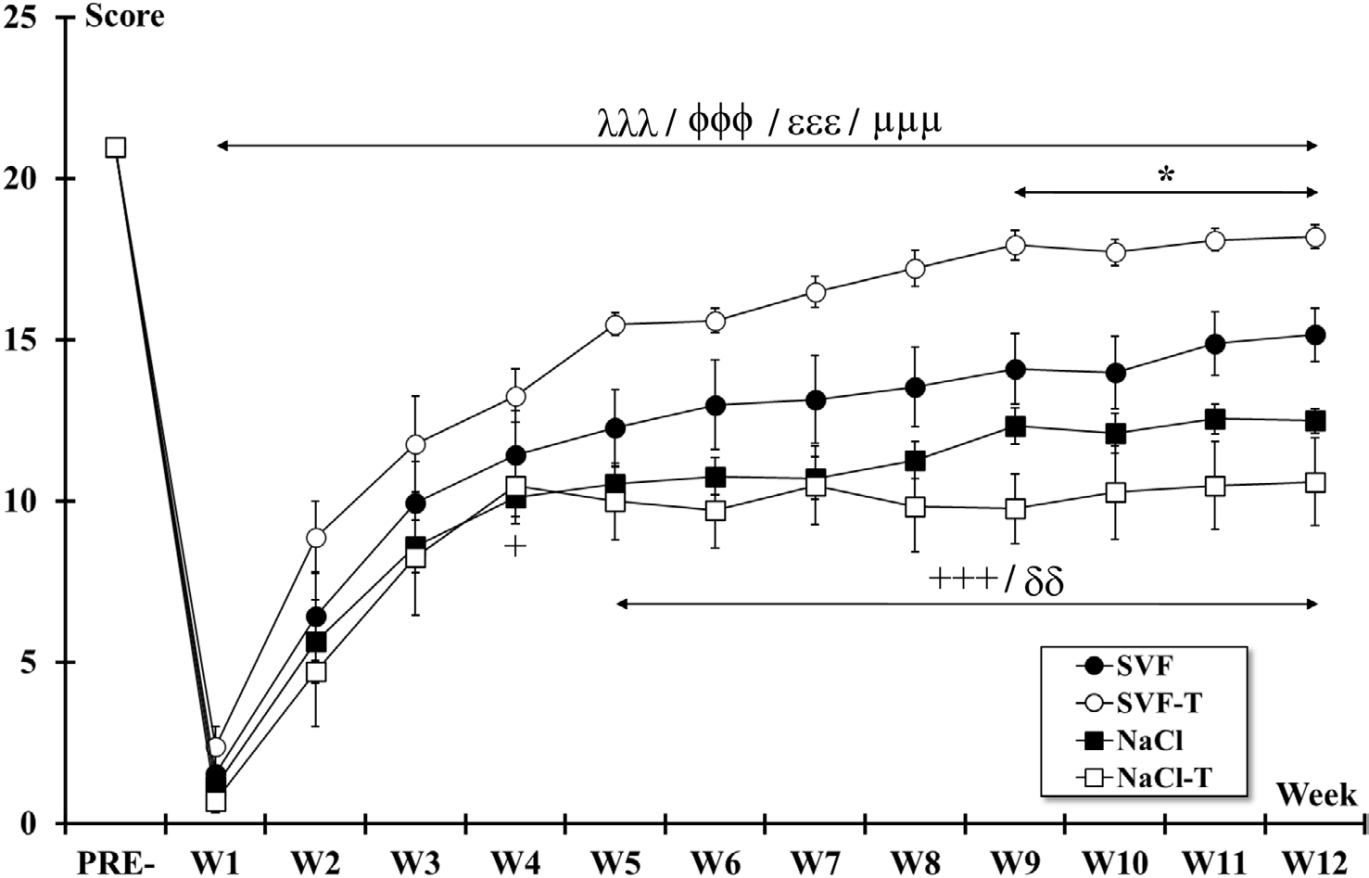
BBB locomotor rating scale. Following spinal cord contusion, the locomotor score of each group demonstrated a significant decrease, followed by a gradual recovery until W12. From W9, the SVF‐T group exhibited a significantly higher locomotor score than the SVF group. Additionally, the score of the SVF‐T group was also significantly higher than the NaCl group from W4 to W12 and the NaCl‐T group from W5 to W12. No significant differences were observed between the SVF, NaCl, and NaCl‐T groups during the 12‐week follow‐up period. The scores of each group remained significantly below their respective −PRE values over the entire study period. Significant difference is indicated by a “*” (SVF vs. SVF‐T), “+” (NaCl vs. SVF‐T), “δ” (NaCl‐T vs. SVF‐T), “λ” (SVF, PRE‐ vs. post‐injury), “Φ” (SVF‐T, PRE‐ vs. post‐injury), “ε” (NaCl, PRE‐ vs. post‐injury) and “μ” (NaCl‐T, PRE‐ vs. post‐injury). One symbol, *p* < 0.05, two symbols, *p* < 0.01 and three symbols, *p* < 0.001. Two‐way ANOVA, *n* = 9 per group.

All animals exhibited a significant (group × time interaction: *F*_(36,384)_ = 3.833; *p* < 0.001) drop in BBB scores the first week (W1) post‐injury compared to the reference values obtained before the injury (PRE‐), indicating a loss of locomotor function. At W1, the comparison between the groups showed no significant difference in the score. Then, the BBB score gradually increased over the following 11 weeks, except for the NaCl‐T group in which BBB score reached a plateau from the W4. At the 12th week, the scores were 12.50 ± 0.37 and 10.61 ± 1.36 (intermediate stage: intervals of uncoordinated stepping) for the NaCl and NaCl‐T groups, respectively, and 15.17 ± 0.83 and 18.22 ± 0.27 (late stage: forelimb and hindlimb coordination with weight support) for the SVF and SVF‐T groups, respectively. Locomotor recoveries were significantly higher for the SVF‐T group from W4 to W12 than the NaCl group (W4: *p* < 0.05; W5 to W12: *p* < 0.001), and from W5 to W12 than the NaCl‐T group (*p* < 0.01). Moreover, the SVF‐T group showed significantly (*p* < 0.05) higher locomotor recoveries than the SVF group from W9 to W12. No difference was found in BBB scores between the SVF, NaCl, and NaCl‐T groups during the 12 weeks of follow‐up. Finally, despite recovery, scores of all groups remained lower than the PRE‐ values (Figure 2).

#### 3.2.2 Ladder Climbing Test

**FIGURE 3:**
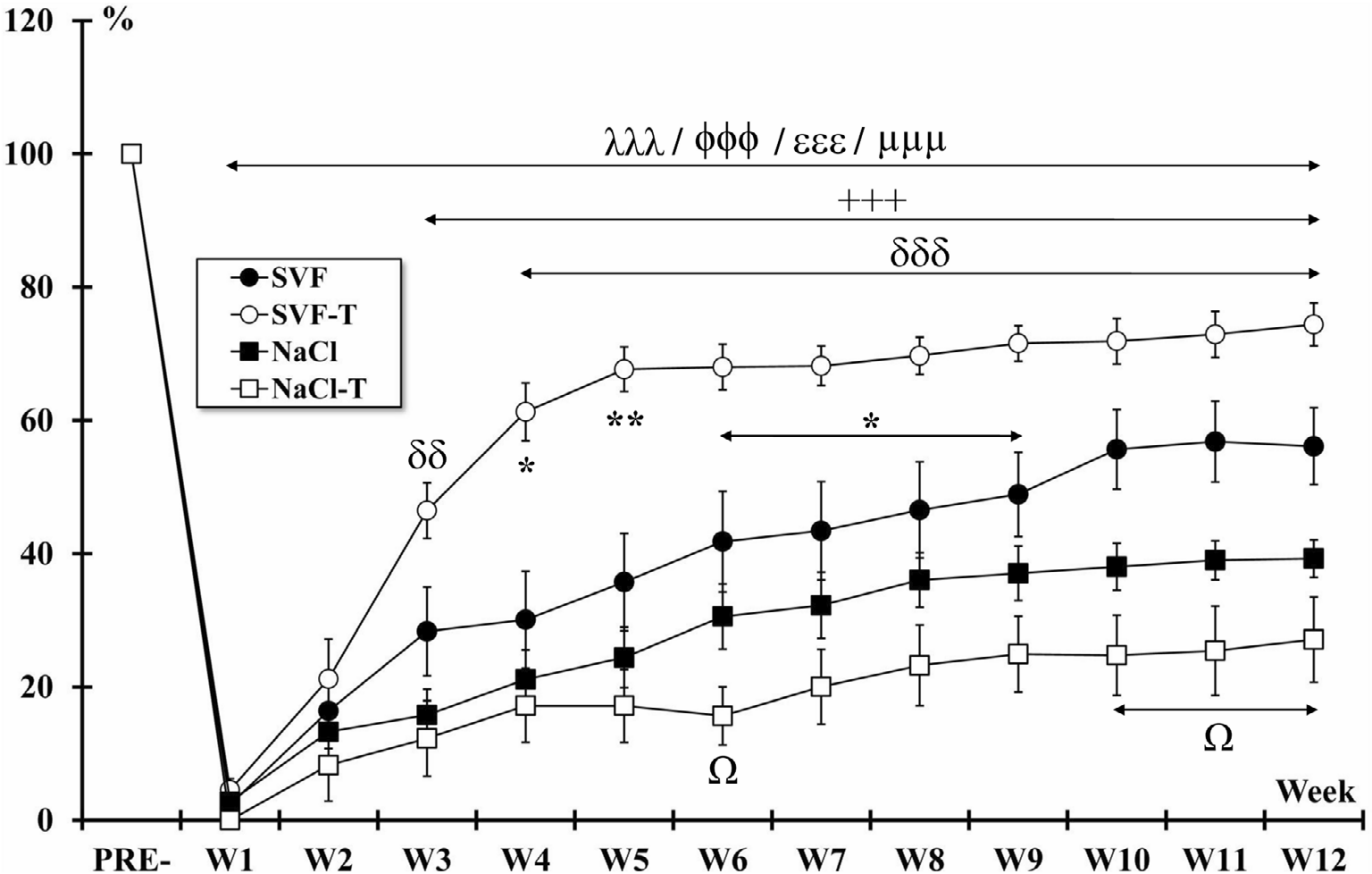
Ladder climbing test. The score is expressed as a percentage of the baseline score measurement at week 0 (PRE‐). In each group, following spinal cord contusion, the climbing score significantly dropped, and then a slow recovery was observed until W12 in SVF, NaCl and NaCl‐T groups. The SVF‐T group exhibited a rapid increase in climbing score, which became significantly higher than the NaCl group from W3 and the NaCl‐T group from W4. The climbing score of the SVF‐T group was also significantly higher than the SVF group from W4 to W9. The score of the SVF group was significantly higher than the NaCl‐T group at W6 and from W10 to W12. The climbing scores of each group remained significantly below their respective −PRE values. Significant difference is indicated by a “*” (SVF vs. SVF‐T), “+” (NaCl vs. SVF‐T); “δ” (NaCl‐T vs. SVF‐T); “Ω” (NaCl‐T vs. SVF); “λ” (SVF, PRE‐ vs. post‐injury), “Φ” (SVF‐T, PRE‐ vs. post‐injury), “ε” (NaCl, PRE‐ vs. post‐injury) and “μ” (NaCl‐T, PRE‐ vs. post‐injury). One symbol, *p* < 0.05, two symbols, *p* < 0.01 and three symbols, *p* < 0.001. Two‐way ANOVA, *n* = 9 per group.

The analysis of the ladder climbing test over the 12 weeks after surgery showed a significant (group × time interaction: *F*_(36,384)_ = 9.484; *p* < 0.001) drop in score in all groups 1 week (W1) after surgery compared to the PRE values, indicating a loss of sensory and motor function. At W1, the comparison between the groups showed no significant difference in the score. Data analysis indicated that the ladder climbing scores slowly increased over the following 11 weeks, and reached 59.45% ± 5.32%, 39.99% ± 3.10% and 25.00% ± 6.85% at W12 for SVF, NaCl and NaCl‐T groups, respectively. Intergroup comparison showed that the scores in the SVF‐T group increased rapidly from W1 to W5 and then stabilized until W12 (W12: 73.16% ± 4.10%). Scores in the SVF‐T group were significantly (*p* < 0.001) higher than those in the NaCl group from W3 to W12, than those in the NaCl‐T group from W4 (*p* < 0.01) to W12 (*p* < 0.001), and then those in the SVF group at W4 (*p* < 0.05), W5 (*p* < 0.01), and W6 (*p* < 0.05), and from W6 to W9 (*p* < 0.05). In addition, there was a significant difference (*p* < 0.05) between the NaCl‐T group and the SVF group at W6 and from W10 to W12. However, no difference was reported between the SVF and NaCl groups and between NaCl and NaCl‐T groups during the 12 weeks of follow‐up. Finally, throughout the study period, scores of all groups remained lower than the PRE‐ values (Figure 3).

#### 3.2.3 Gait Analysis

The regulatory index observed at the end of the W12 post‐surgery was 100% in the SVF and SVF‐T groups and lower in the NaCl (77.78% ± 9.68%) and NaCl‐T (82.22% ± 9.09%) groups.

**FIGURE 4:**
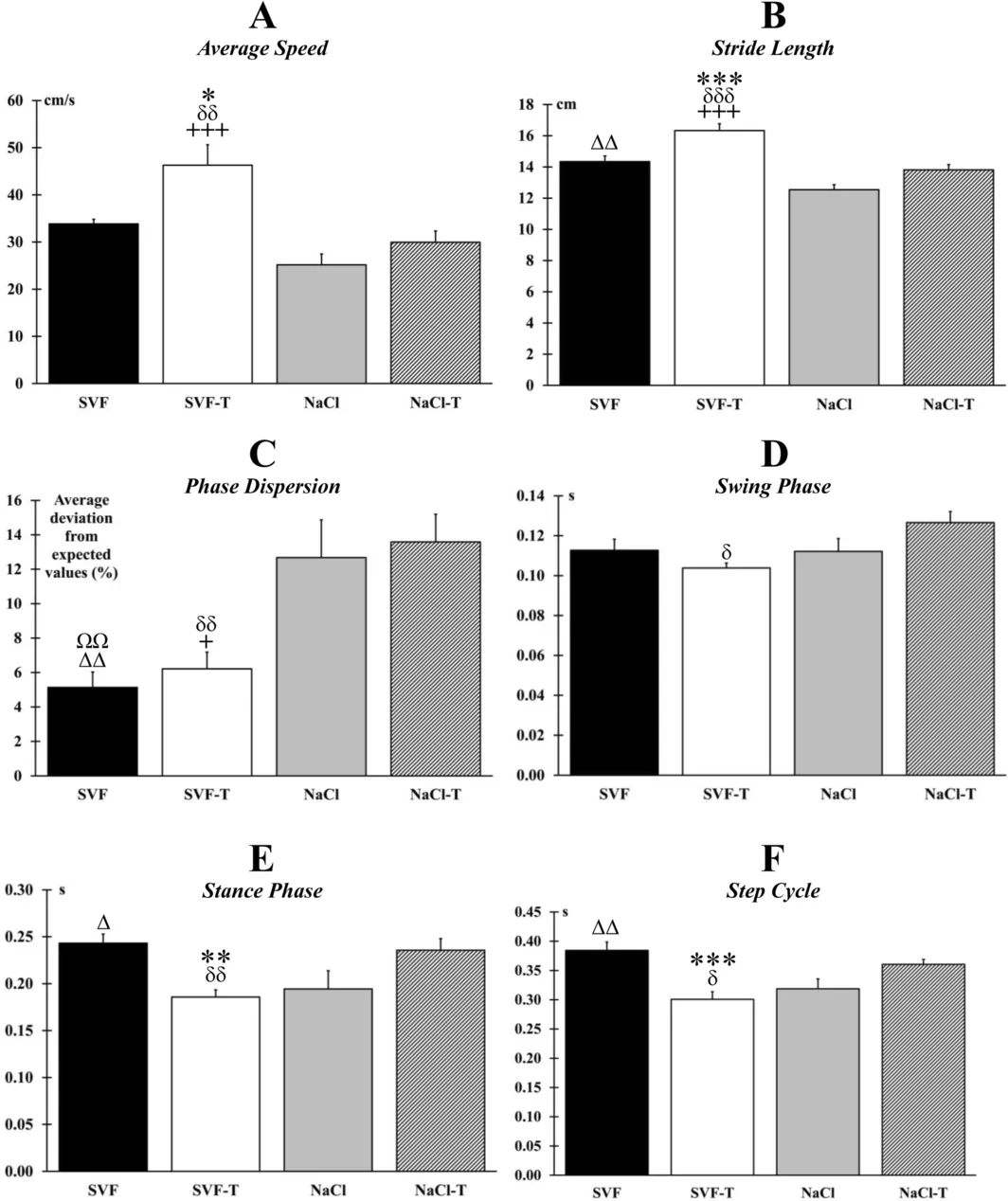
Gait analysis parameters. (A) Average speed. The average speed of passage over the glass plate was significantly higher in the SVF‐T group than in the other three groups. No difference was observed between the SVF, NaCl and NaCl‐T groups. (B) Stride length. The average stride length between hindlimbs was significantly increased in the SVF‐T group compared with the other three groups. The SVF group had significantly longer stride length than the NaCl group. (C) Phase dispersion. The average deviation of the hindlimbs from the expected value of 50% was significantly reduced in animals in the SVF and SVF‐T groups compared with the NaCl and NaCl‐T groups. (D) Swing phase. The average time of the swing phase between hindlimbs was significantly reduced in the SVF‐T group compared with the NaCl‐T group. (E) Stance phase. The average stance phase between hindlimbs showed a significant reduction in the SVF‐T group compared to the SVF and NaCl‐T groups. The stance phase of the NaCl group was also found to be lower than the SVF group. (F) Step cycle. The average step cycle between hindlimbs showed a significant reduction in the SVF‐T and NaCl groups compared to the SVF group. The time of step cycle of the SVF‐T group was also found to be lower than the NaCl‐T group. Significant difference is indicated by a “*” (SVF vs. SVF‐T), “+” (NaCl vs. SVF‐T), “δ” (NaCl‐T vs. SVF‐T), “Δ” (SVF vs. NaCl) and “Ω” (NaCl‐T vs. SVF). One symbol, *p* < 0.05, two symbols, *p* < 0.01 and three symbols, *p* < 0.001. One‐way ANOVA, *n* = 9 per group.

The results of the one‐way ANOVA (*F*_(3,32)_ = 10.63; *p* < 0.0001) also indicated that animals from the SVF‐T group (46.29 ± 4.34 cm/s) showed a significant higher mean crossing speed on the glass plate than the SVF (33.85 ± 0.96 cm/s; *p* < 0.05), NaCl (25.16 ± 2.30 cm/s; *p* < 0.001) and NaCl‐T (29.94 ± 2.39 cm/s; *p* < 0.01) groups. There was no difference between the SVF, NaCl and NaCl‐T groups (Figure 4A). Data also showed that the stride length (*F*_(3,62)_ = 18.82; *p* < 0.0001) was significantly increased in SVF‐T group (16.35 ± 0.36 cm; *p* < 0.001) compared to the SVF (14.35 ± 0.33 cm), NaCl (12.68 ± 0.26 cm) and NaCl‐T (14.50 ± 0.33 cm) groups. Moreover, the stride length was significantly reduced in the NaCl group compared to the SVF (*p* < 0.01) group, while no difference was found between the SVF and NaCl‐T groups, and between NaCl and NaCl‐T groups (Figure 4B). The results (*F*_(3,31)_ = 7.866; *p* = 0.0005) also showed that animals in the NaCl‐T groups (13.59% ± 1.61%) exhibited a significant increase (*p* < 0.01) in phase dispersion compared to the SVF (5.49% ± 0.87%) and SVF‐T (6.22% ± 0.97%) groups. The phase dispersion was also higher in NaCl group (12.67% ± 2.19%) than in SVF (*p* < 0.01) and SVF‐T (*p* < 0.05) groups. No difference was found between NaCl and NaCl‐T groups, and between SVF and SVF‐T groups (Figure 4C). Moreover, the swing phase duration (*F*_(3,54)_ = 3.256; *p* = 0.0285) was significantly (*p* < 0.05) reduced in the SVF‐T group (0.10 ± 0.01 s) compared to the NaCl‐T group (0.13 ± 0.01 s) but did not showed difference between the other groups (Figure 4D). The stance phase duration (*F*_(3,51)_ = 7.599; *p* = 0.0003) between the hindlimbs showed a significant reduction in the SVF‐T (0.19 ± 0.01 s; *p* < 0.01) and NaCl (0.18 ± 0.01 s; *p* < 0.05) groups compared to the SVF group (0.24 ± 0.01 s). The stance phase duration of the SVF‐T group was also significantly lower than that of the NaCl‐T group (0.27 ± 0.02 s; *p* < 0.05). No difference was observed between the SVF‐T and NaCl groups, between the SVF and NaCl‐T groups, and between the NaCl and NaCl‐T groups (Figure 4E). Finally, the SVF group (0.38 ± 0.01 s) exhibited a significantly increase in the step cycle time (*F*_(3,54)_ = 8.802; *p* < 0.0001) compared to the SVF‐T (0.30 ± 0.01 s, *p* < 0.001) and the NaCl (0.32 ± 0.02 s; *p* < 0.01) groups. Moreover, the step cycle time was significantly higher in the NaCl‐T group (0.36 ± 0.01 s; *p* < 0.05) compared to the SVF‐T group. No difference was observed between the SVF‐T and NaCl groups, between the SVF and NaCl‐T groups, and between the NaCl and NaCl‐T groups (Figure 4F).

The results of the analysis of the frequency of the regular step pattern revealed a majority of the “alternate” step pattern among all groups (SVF: 84.44%; SVF‐T: 97.78%; NaCl: 71.43%; NaCl‐T: 68.89%). Physiologically, healthy rats generally exhibit step patterns called “cruciate” and “rotate,” which are considered normal step patterns. Here, these two step patterns represented 15.55% for the SVF group, 2.22% for the SVF‐T group, and 22.22% and 13.33% for the NaCl and NaCl‐T groups, respectively. Moreover, the animals in the NaCl and NaCl‐T groups exhibited 22.22% and 17.78% of step patterns considered abnormal, and 0% for the other two groups.

### 3.3 Electrophysiological Recordings

**FIGURE 5:**
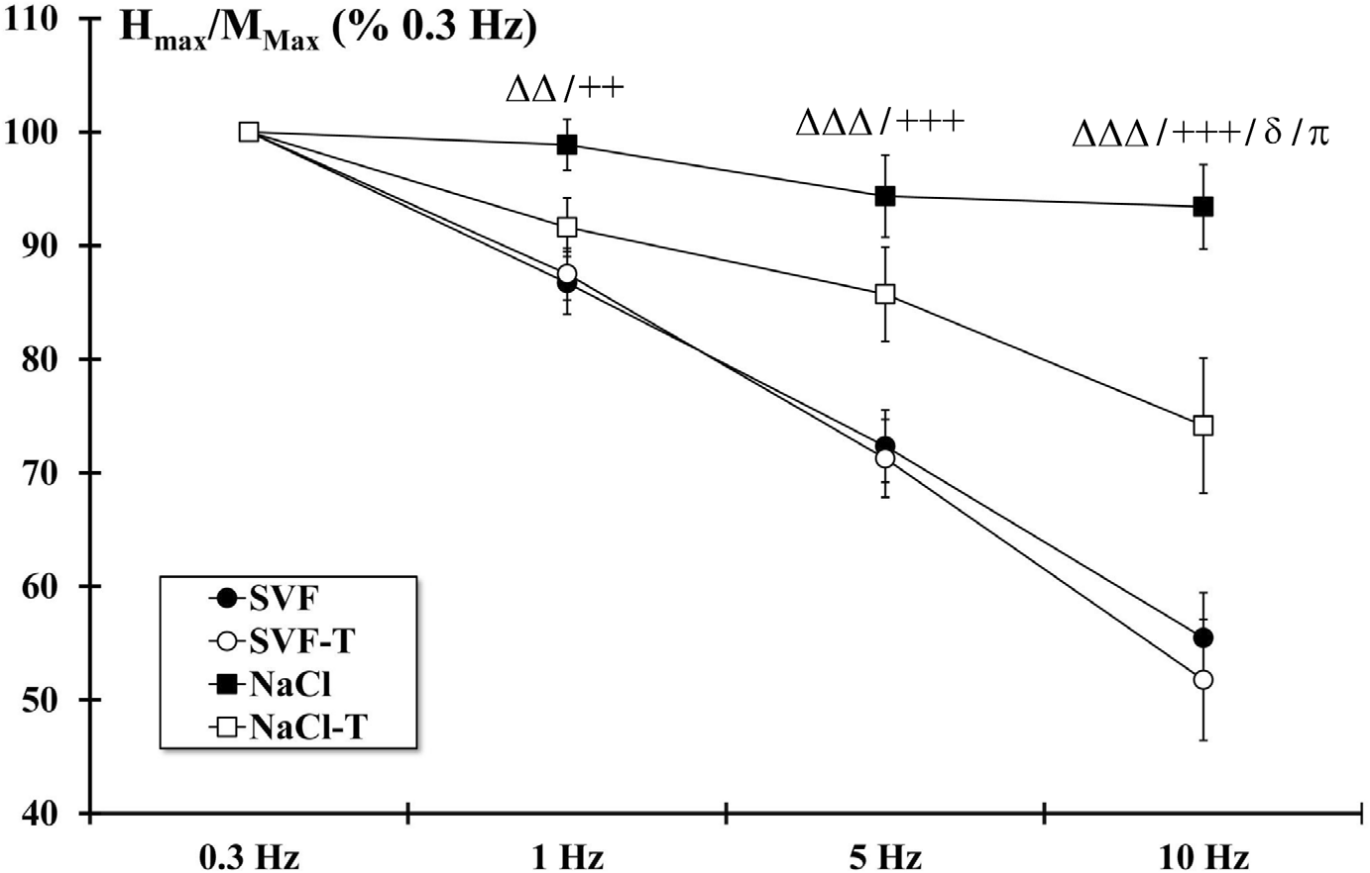
H‐refex rate sensitivity. The H_max_/M_max_ ratio significantly decreased with increasing stimulation frequency in the SVF, SVF‐T and NaCl‐T groups compared to ratio measured at 0.3 Hz. Intergroup comparison showed that the ratios of the SVF and SVT were lower than ratios of NaCl group for 1, 5 and 10 Hz. At 10 Hz, significant differences were also observed between SVF‐T and NaCl‐T groups, and between NaCl and NaCl‐T groups. For a given frequency, the significant difference is indicated by a “Δ” (SVF vs. NaCl), “+” (NaCl vs. SVF‐T), “δ” (NaCl‐T vs. SVF‐T) and “π” (NaCl vs. NaCl‐T). One symbol, *p* < 0.05, two symbols, *p* < 0.01 and three symbols, *p* < 0.001. Two‐way ANOVA, *n* = 9 per group.

The baseline H_max_/M_max_ ratios measured at a stimulation frequency of 0.3 Hz were 0.40 ± 0.03, 0.37 ± 0.03, 0.38 ± 0.04 and 0.38 ± 0.04 for the SVF, SVF‐T, NaCl and NaCl‐T groups, respectively. At this frequency, no difference was found between the groups. The H_max_/M_max_ ratio decreased with increasing stimulation frequency in the SVF, SVF‐T and NaCl‐T groups (group × frequency interaction: *F*_(9,204)_ = 10.57; *p* < 0.0001). Indeed, the H_max_/M_max_ ratio values at 1, 5, and 10 Hz were, respectively, (1) 86.70% ± 2.75% (*p* < 0.001), 72.33% ± 3.17% (*p* < 0.001) and 55.46% ± 3.97% (*p* < 0.001) of the ratio measured at the baseline stimulation in the SVF group, (2) 87.49% ± 2.27% (*p* < 0.001), 71.26% ± 3.42% (*p* < 0.001) and 51.76% ± 5.33% (*p* < 0.001) in the SVF‐T group and (3) 91.61% ± 2.58% (*p* < 0.05), 85.72% ± 4.16% (*p* < 0.05) and 74.14% ± 5.96% (*p* < 0.01) in the NaCl‐T group. In the NaCl group, the H_max_/M_max_ *ratios* remained unchanged with increasing frequency (1 Hz: 98.86% ± 2.24%, 5 Hz: 94.35% ± 3.62% and 10 Hz: 93.42% ± 3.71%). Intergroup comparison revealed that in the SVF and SVF‐T groups, the *ratios* were statistically lower than the NaCl group at all three frequencies: 1 Hz (*p* < 0.01), 5 Hz (*p* < 0.001), and 10 Hz (*p* < 0.001). Moreover, analysis of the H_max_/M_max_ ratio at 10 Hz indicated a significant difference between the SVF‐T and the NaCl‐T groups (*p* < 0.05), and between the NaCl‐T and the NaCl groups (*p* < 0.05). No significant difference was found between the SVF and SVF‐T groups regarding the depression of the H‐reflex response at any of the tested frequencies. All ratio values and comparisons are presented in Figure 5.

**FIGURE 6:**
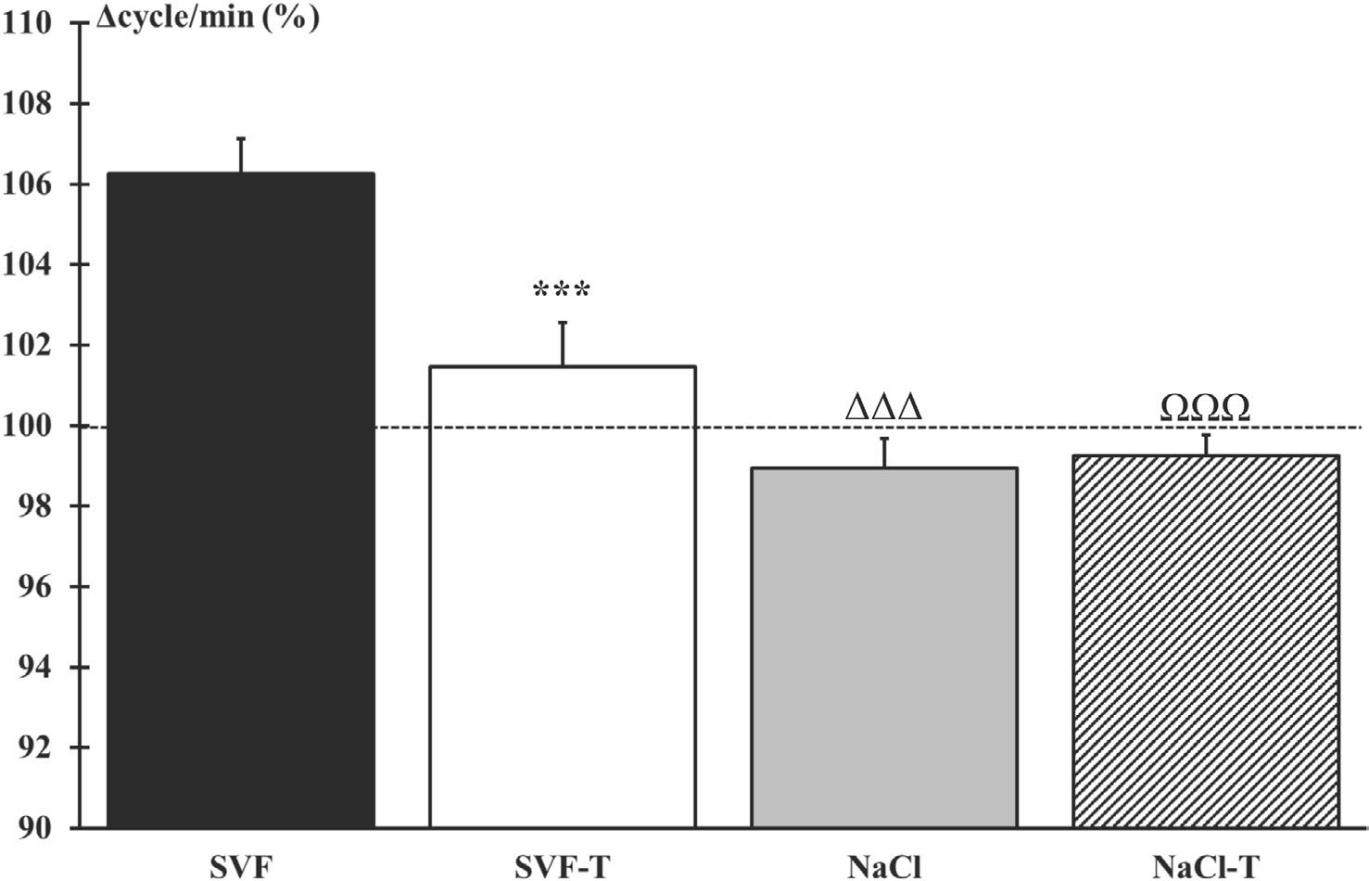
Ventilatory adjustment to 3‐min EIF. Ventilatory frequency was significantly higher in the SVF group compared to the SVF‐T, NaCl, and NaCl‐T groups during a 3‐min muscle stimulation of the tibialis anterior muscle and under local regional circulatory occlusion. No significant difference was observed between the SVF‐T, NaCl, and NaCl‐T groups. Significant difference is indicated by a “*” (SVF vs. SVF‐T), “Δ” (SVF vs. NaCl), and “Ω” (NaCl‐T vs. SVF). Three symbols, *p* < 0.001. One‐way ANOVA, *n* = 9 per group.

The one‐way ANOVA of ventilatory frequency (*F*_(3,71)_ = 18.43; *p* < 0.0001) after activation of metabosensitive afferent fibers by *Tibialis anterior* muscle repetitive stimulation under regional circulatory occlusion was significantly (*p* < 0.001) increased in SVF (106.26% ± 0.87%) group. No increase in ventilatory frequency was noted in SVF‐T (101.46% ± 0.85%), NaCl (98.94% ± 0.75%) and NaCl‐T (99.25% ± 0.52%) groups. Intergroup comparison indicated a significant (*p* < 0.001) difference between the SVF and the three other groups. No difference was found between SVF‐T, NaCl‐T and NaCl groups (Figure 6).

## 4 Discussion

In a previous study, we demonstrated the therapeutic potential of autologous SVF to reduce endogenous inflammation and to improve functional recovery in animals immediately treated after a thoracic spinal cord contusion (Ertlen et al. 2024). Complementary electrophysiological analyses showed that subdural injection of autologous SVF at the injury site restored functionality of the segmental sensorimotor loop and communication between the sub‐lesion site and supraspinal centers (Ertlen et al. 2024). Building on our previous findings, the present study aimed to determine whether combining autologous SVF transplantation with treadmill training could further enhance functional recovery after severe thoracic SCI.

### 4.1 The Combined Therapy Improves Functional Recovery After SCI

As previously described, in this study, the BBB and ladder climbing tests revealed a significant decrease in locomotor function in all animal groups 1 week after injury, indicating complete hindlimb paralysis with our contusion model (Krishna et al. 2013; Scheff et al. 2003). A partial spontaneous recovery was observed over time in all groups, in agreement with the limited endogenous repair capacity that follows SCI (Raineteau and Schwab 2001). Then, the results of both tests showed that training potentiated the effects of SVF by further improving locomotor recovery in the SVF‐T group compared to the other groups. Surprisingly, analysis of behavioral tests results showed no significant difference in locomotor recovery in the NaCl and NaCl‐T groups during the 12 weeks following injury.

Gait analysis confirmed these behavioral findings by showing faster locomotion, longer strides, and a shorter gait cycle in the SVF‐T group. Together, these changes indicate a more efficient locomotor pattern, suggesting that the combined treatment improved the execution of weight‐supported stepping rather than locomotor coordination itself. Indeed, phase dispersion remained unchanged between the SVF and SVF‐T groups, indicating that interlimb coordination was already largely recovered with SVF treatment alone. Thus, in our model, treadmill training appears to have optimized locomotor performance by increasing walking efficiency rather than by further reorganizing the coordination pattern, which is consistent with the literature (Hubli and Dietz 2013; Lam et al. 2007).

Consistent with our previous study, SVF treatment restored RDD of the H‐reflex, suggesting a partial normalization of spinal reflex excitability and inhibitory sensorimotor circuitry following SCI, regardless of the addition of treadmill training. In contrast, RDD was not restored in the NaCl and NaCl‐T groups, except for a modest improvement at 10 Hz in the NaCl‐T group. Interestingly, this partial electrophysiological improvement was not associated with better locomotor performance in the BBB or ladder climbing tests. As this apparent discrepancy raises important questions regarding the respective functional significance of electrophysiological and behavioral outcomes, it is discussed in a dedicated section below. It is also important to note that although anesthetics such as ketamine and isoflurane can influence spinal excitability—potentially dampening baseline H‐reflex amplitudes or altering RDD under specific stimulation protocols (Ho and Waite 2002; Wieters et al. 2022), all animals in our study were anesthetized with the same agents. Therefore, any possible influence of anesthesia would be consistent across groups and would not affect the interpretation of treatment efficacy.

EIF allows evaluating the functional transmission between sublesional spinal circuits and pontomedullary respiratory centers (Darques and Jammes 1997; Decherchi et al. 2007; McCloskey and Mitchell 1972). In the present study, no ventilatory adjustment was observed following EIF in the NaCl group, whereas the SVF group exhibited a significant increase in ventilation after repetitive muscle stimulation. These findings are consistent with our previous study and suggest that autologous SVF transplantation improves the functional transmission of the neural pathways involved in ventilatory regulation following SCI. Unexpectedly, treadmill training did not modify ventilatory adjustments in either the NaCl‐T or SVF‐T groups. Although exercise training has been reported to improve respiratory function after SCI through neural and muscular adaptations (Lemos et al. 2020), these adaptations may also increase respiratory efficiency, thereby reducing the magnitude of ventilatory responses to standardized muscle stimulation (Tang et al. 2022). In addition, endurance training may induce changes in muscle phenotype (Marqueste et al. 2004), potentially modifying the activation of metabosensitive afferents during EIF. The mechanisms underlying the absence of training‐induced ventilatory adaptations remain unclear and warrant further investigation.

Finally, although the literature indicates that repetitive stimulation of a skeletal muscle using the parameters we employed and under regional circulatory occlusion leads to the release of muscle metabolites, which in turn results exclusively in the activation of metabosensitive afferents (Darques and Jammes 1997; Decherchi et al. 2007) because these ascending pathways are not fully described, we cannot rule out the possibility that collateral branches of the nerve fibers in these pathways may connect to various neurons in the spinal cord and trigger their activation in various spinal segments. As this has not yet been described, it is difficult to know exactly what is happening in the spinal cord apart from the activation of the pontobulbar centers and adjustment of the ventilatory rhythm to EIF.

Taken together, these complementary behavioral and electrophysiological assessments indicate that combining SVF transplantation with treadmill training provides the greatest overall functional benefit after severe SCI. While treadmill training mainly enhanced locomotor performance, SVF transplantation appears to have facilitated the restoration of spinal inhibitory circuitry and ventilatory adjustments. These findings are consistent with previous studies reporting that combining stem cell transplantation with treadmill rehabilitation leads to greater functional recovery than either intervention alone after SCI (Keikhaei et al. 2023; Takahashi et al. 2023; Tashiro et al. 2016). Although these studies used different cell types and rehabilitation paradigms, they collectively suggest that physical rehabilitation can potentiate the therapeutic effects of cell‐based therapies. Our results extend these observations by showing that a similar beneficial interaction can also be achieved using autologous SVF transplantation.

The biological mechanisms underlying this interaction remain to be fully elucidated but are likely to involve complementary effects of SVF transplantation and activity‐dependent plasticity induced by locomotor training. These potential mechanisms are discussed in the following section.

### 4.2 Treadmill Training Was Not Sufficient to Improve Behavioral Recovery

One notable finding of the present study is that treadmill training alone did not produce a detectable improvement in BBB or ladder climbing performance compared with untreated animals. This result contrasts with the majority of experimental studies reporting beneficial effects of locomotor training after SCI. For example, Sun et al. observed significant improvements in BBB scores as early as 3 weeks after a moderate T10 contusion, while Jung et al. reported enhanced locomotor recovery from week 4 using a similar treadmill protocol (Jung et al. 2016; Sun et al. 2013). Likewise, Wang et al. described significant locomotor improvements after treadmill training following a severe thoracic contusion, although these effects became apparent only from week 9 after injury (Wang et al. 2015). Collectively, these studies indicate that treadmill training generally promotes activity‐dependent plasticity and functional recovery after SCI. Nevertheless, our findings are in agreement with those of Alluin et al. who also reported no additional behavioral benefit of treadmill training. The authors proposed that if the number of spared fibers was sufficient to induce locomotion, then self‐training occurring spontaneously in the cage could be responsible for the locomotor improvement beyond which further external training would not be effective (Alluin et al. 2011). A similar explanation may also apply in the present study.

However, the effectiveness of treadmill‐based rehabilitation depends on numerous experimental variables, including the severity and level of the lesion, training onset, session duration, intensity, progression, body‐weight support, and the amount of active stepping performed during each session (Lam et al. 2007; Smith and Knikou 2016). In our severe T10 contusion model, several animals in the NaCl‐T group remained unable to support their body weight throughout the rehabilitation period and therefore required continuous manual assistance. Consequently, the number and quality of effective weight‐supported stepping cycles were likely reduced, limiting the induction of activity‐dependent plasticity and, therefore, the efficacy of treadmill training alone. In addition, the severity of the lesion may have restricted the capacity of rehabilitation to induce detectable behavioral improvements within the 12‐week follow‐up period. Taken together, these observations suggest that the treadmill protocol used here was sufficient to engage the animals in rehabilitation but may not have been optimal as a stand‐alone intervention in this severe contusion model.

The electrophysiological findings also help frame this result more cautiously. The NaCl‐T group showed changes in H‐reflex modulation that were not paralleled by comparable improvements in BBB or ladder scores, indicating that electrophysiological and behavioral recovery do not necessarily evolve in parallel. This dissociation is not surprising, because reflex measures and locomotor tests capture different levels of sensorimotor organization. In this context, the H‐reflex primarily reflects spinal excitability and sensorimotor integration (Hultborn et al. 1996; Little and Halar 1985), whereas BBB and ladder climbing assess more integrated locomotor performance requiring the coordinated contribution of multiple descending, propriospinal, sensory, and motor pathways (Basso et al. 1995; Metz and Whishaw 2009; Semler et al. 2011).

Taken together, these findings suggest that, under our experimental conditions, treadmill training alone did not constitute a sufficiently powerful therapeutic intervention to improve locomotor behavior. However, when combined with SVF transplantation, rehabilitation likely potentiated the beneficial effects of cell therapy by enabling animals to better engage in activity‐dependent plasticity, resulting in greater functional recovery than either treatment alone.

### 4.3 Possible Mechanisms Underlying the Combined Effect

Although the present study was not designed to investigate the biological mechanisms underlying the functional improvements observed after the combined therapy, several complementary processes may explain the beneficial interaction between autologous SVF transplantation and treadmill training. The functional improvements observed in the SVF‐T group most likely arise from the interaction between the biological effects of SVF transplantation and the activity‐dependent adaptations induced by rehabilitation rather than from either intervention acting independently. These hypotheses should, however, be interpreted with caution, as they were not directly investigated in the present work and will require dedicated histological and molecular studies.

SVF is a heterogeneous cellular product composed of adipose‐derived stem cells (ADSCs), endothelial progenitor cells, pericytes, macrophages, lymphocytes, and other stromal cells that collectively exert neuroprotective, angiogenic, immunomodulatory, and regenerative effects, mainly through paracrine signaling (Aubertin et al. 2021; Gandolfi et al. 2023). Previous experimental studies have shown that ADSCs transplantation after SCI can reduce secondary tissue damage, preserve spinal tissue, promote angiogenesis, attenuate neuroinflammation, and support axonal sprouting, ultimately contributing to functional recovery (Gao et al. 2019; Liang et al. 2014; Ohta et al. 2018; Pang et al. 2021; Rafiei Alavi et al. 2021).

Conversely, treadmill training is known to promote activity‐dependent plasticity within spinal and supraspinal locomotor networks. Repetitive locomotor activity increases the expression of neurotrophic factors such as brain‐derived neurotrophic factor (BDNF) and glial cell line‐derived neurotrophic factor (GDNF), enhances synaptic plasticity, and facilitates the reorganization of sensorimotor circuits after SCI (Jung et al. 2014; Kiss Bimbova et al. 2022; Lewis et al. 2022; Tashiro et al. 2015). These neurotrophic factors are known to facilitate synaptic strengthening, neuronal survival, and axonal sprouting, all of which are critical for activity‐dependent functional recovery after SCI. Rehabilitation has also been associated with cortical reorganization and strengthening of spared neuronal pathways, thereby improving the efficiency of locomotor control (Fouad et al. 2001; Hamers et al. 2001; Martinez et al. 2009). Collectively, these adaptations may increase the capacity of spared neural circuits to generate functional locomotor outputs during rehabilitation.

The greater functional recovery observed in the SVF‐T group therefore likely results from complementary rather than independent actions of these two therapeutic approaches. We hypothesize that SVF transplantation may create a more permissive microenvironment by limiting secondary injury and promoting tissue preservation, while treadmill training provides the activity‐dependent stimulation required to reinforce synaptic plasticity and optimize the functional use of spared or newly established neural connections. In this context, the improved locomotor abilities induced by SVF may also have enabled animals to engage more effectively in rehabilitation, thereby further enhancing activity‐dependent plasticity.

This interpretation aligns with previous works showing that combining cell‐based therapy with locomotor rehabilitation not only improves functional outcomes after SCI, but also promotes greater tissue preservation, reduced lesion and cavity formation, increased angiogenesis, attenuation of macrophage infiltration and neuroinflammation, and enhanced neuronal plasticity following combined therapy (Keikhaei et al. 2023; Takahashi et al. 2023; Tashiro et al. 2016). Collectively, these observations support the hypothesis that rehabilitation may both improve the functional use of spared neural circuits and enhance the therapeutic microenvironment created by transplanted cells. However, because the present study did not include histological, molecular, or cellular analyses, we cannot determine which of these mechanisms contributed to the functional improvements observed.

### 4.4 Limitations and Future Directions

The present study demonstrates that combining autologous SVF transplantation with treadmill training enhances functional recovery after severe thoracic SCI. Although these findings provide a strong proof‐of‐concept for this combinatorial strategy, several questions remain to be addressed to further optimize its therapeutic potential and better understand the mechanisms involved.

First, the present work focused on behavioral, gait, and electrophysiological outcomes without investigating the associated biological changes within the injured spinal cord. Future studies combining histological and molecular analyses will be essential to determine whether the functional improvements observed are associated with greater tissue preservation, axonal remodeling, modulation of the inflammatory response, angiogenesis, and long‐term survival or integration of transplanted SVF cells. Such analyses would also help verify lesion consistency across experimental groups and clarify the biological mechanisms underlying recovery.

Second, only one treadmill rehabilitation protocol was evaluated in the present study. Although this protocol successfully potentiated the therapeutic effects of SVF transplantation, it did not significantly improve behavioral recovery when applied alone. Future studies should therefore investigate whether modifying rehabilitation parameters—including the timing of rehabilitation onset, training duration, intensity, progression, body‐weight support, or alternative rehabilitation paradigms—could further enhance the efficacy of both rehabilitation alone and its combination with SVF transplantation.

Third, a slight difference in early functional performance was observed between the SVF and SVF‐T groups before the onset of training. Although this difference was not statistically significant, it may reflect inter‐individual variability in lesion severity, early post‐operative recovery, or graft integration. Because no histological quantification of lesion severity was available, we cannot exclude that inter‐individual variability contributed to the small baseline difference observed between SVF and SVF‐T groups.

Overall, these limitations highlight the need for further mechanistic and comparative studies, while the present data already support the functional value of combining SVF transplantation with rehabilitation.

## 5 Conclusion

The present findings demonstrate that combining autologous SVF transplantation with treadmill training improves functional recovery after severe thoracic SCI. While treadmill training alone did not significantly enhance behavioral recovery, it potentiated the beneficial effects of SVF, supporting the concept that rehabilitation and cell therapy exert complementary actions. Further mechanistic and histological studies will be required to identify the biological processes underlying this interaction and to optimize rehabilitation protocols, with the ultimate goal of evaluating its possible integration into therapeutic protocols for individuals with SCI.

## Author Contributions

Conceptualization, C.E., M.B., P.D., T.M.; methodology, C.E., M.B., P.D., T.M., J.‐M.B.; software, T.C.; validation, C.E., M.B., P.D., T.M., J.‐M.B., M.S.; formal analysis, C.E., M.B., P.D., T.M.; investigation, C.E., M.B., P.D., T.M.; resources, N.S., P.D.; data curation, C.E., M.B.; writing – original draft preparation, C.E., M.B., P.D., T.M.; writing – review and editing, C.E., T.C.; M.B., P.D., T.M., M.S.; visualization, C.E., P.D.; supervision, P.D., T.M.; project administration, P.D., T.M., N.S.; funding acquisition, P.D., N.S.; all authors have read and agreed to the published version of the manuscript.

## Funding

This work was supported by public [Aix‐Marseille Université (AMU) and Centre National de la Recherche Scientifique (CNRS)] and by private [Combattre la Paralysie Association] (No. 2022‐2) grants. The funding body played no role in the design of the study and collection, analysis, and interpretation of data and in writing the manuscript.

## Disclosure

Artificial intelligence was not used in any of the stages of this work (experimentation, data collection, analysis, interpretation, visualization, reporting and editing, manuscript writing).

## Consent

The authors have nothing to report.

## Conflicts of Interest

The authors declare no conflicts of interest.

## Supporting information


**Data S1:** Supporting Information.

## Data Availability

All data analyzed during this study are included in this publication. The datasets during and/or analyzed during the current study are available from the corresponding author on reasonable request.
